# SNP@Promoter: a database of human SNPs (Single Nucleotide Polymorphisms) within the putative promoter regions

**DOI:** 10.1186/1471-2105-9-S1-S2

**Published:** 2008-02-13

**Authors:** Byoung-Chul Kim, Woo-Yeon Kim, Daeui Park, Won-Hyong Chung, Kwang-sik Shin, Jong Bhak

**Affiliations:** 1 Korean BioInformation Center (KOBIC), KRIBB, Daejeon 305-806, Korea

## Abstract

**Background:**

Analysis of single nucleotide polymorphism (SNP) is becoming a key research in genomics fields. Many functional analyses of SNPs have been carried out for coding regions and splicing sites that can alter proteins and mRNA splicing. However, SNPs in non-coding regulatory regions can also influence important biological regulation. Presently, there are few databases for SNPs in non-coding regulatory regions.

**Description:**

We identified 488,452 human SNPs in the putative promoter regions that extended from the +5000 bp to -500 bp region of the transcription start sites. Some SNPs occurring in transcription factor (TF) binding sites were also predicted (47,832 SNP; 9.8%). The result is stored in a database: SNP@promoter. Users can search the SNP@Promoter database using three entries: 1) by SNP identifier (rs number from dbSNP), 2) by gene (gene name, gene symbol, refSeq ID), and 3) by disease term. The SNP@Promoter database provides extensive genetic information and graphical views of queried terms.

**Conclusion:**

We present the SNP@Promoter database. It was created in order to predict functional SNPs in putative promoter regions and predicted transcription factor binding sites. SNP@Promoter will help researchers to identify functional SNPs in non-coding regions.

## Background

After finishing the Human Genome Project, biologists' interest has shifted to non-repetitive sequence variants in genome, by far the most common of which are single nucleotide polymorphisms (SNPs). For a variation to be considered an SNP, it must occur in at least 1% of the population. SNPs, which make up about 90% of all human genetic variation, occur every 100 to 300 bases along the 3-billion-base human genome [1,2]. It is generally believed that the complete human sequence will reveal at least a million SNPs of coding regions, including introns and promoters. As a general rule, many SNPs have no effect on cell function, but some SNPs are reported to be highly related to diseases or to influence cells' response to a drug. Although more than 99% of human DNA sequences are the same across all populations, some SNPs can have a major impact on how humans respond to diseases; environmental insults such as bacteria, viruses, toxins, and chemicals; and drugs and other therapies. This makes SNPs of great value for biomedical research and for developing pharmaceutical products and for medical diagnostics.

New bioinformatics tools and public SNP resources for SNP studies, specifically for linkage disequilibrium and disease association studies, will form part of the new scientific landscape [3-9]. These public SNP resources are possible through the large-scale and high-throughput systems to screen SNPs on many individuals. The challenge is to accomplish this while reducing the cost per genotype and required completion time. The public SNP resources are producing information about SNPs which are related to diseases or that modify biological function. Many functional studies of SNPs were focused on SNPs located in coding regions that can influence phenotype by altering the encoded proteins [9,10]. They can also influence premature termination that can cause nonsense-mediated mRNA decay (NMD) [11]. Another function of SNPs is that they affect splice sites which results in alternative splicing [12].

Additionally, there are many SNPs in non-coding regulatory regions. The exact functions of the non-coding regulatory region SNPs are not clear yet. However, some SNPs are predicted to be related to genes by influencing the binding affinity of transcription factors. For example, the G/C polymorphism in the promoter region of the FCGR2B promoter regulates gene expression [13]. -783A/G and -1438A/G polymorphisms in the promoter of HTR2A gene regulate gene expression. -783 G allele and -1438 G allele are known to reduce the binding activity of transcription factors [14]. However, there are no public resources that provide promoter information of SNPs influencing the non-coding regulatory regions in the human genome. The rSNP_Guide system is the only one that has reported SNPs that are related to potential transcription factor candidates among 41 types of known transcription factor binding sites. [15,16]. ORegAnno is focused not on SNP information of the regulatory regions in the human genome but on the registration and validation of SNPs from promoters, transcription factor binding sites, and regulatory variation [17].

SNP@Promoter is a large database that contains various types of information on the location and function for putative promoter regions in the human genome for gene regulation study. In particular, SNP@Promoter provides a platform for biologists including disease associated genes, transcription factor binding sites, and a graphic viewer.

## Methods and results

**Figure 1:**
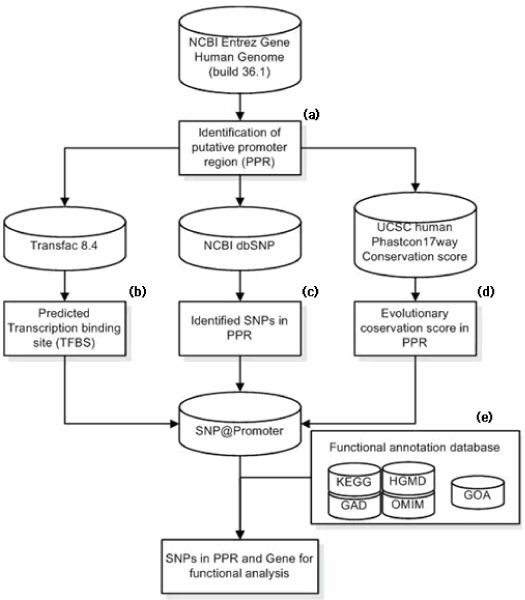
**Flow chart for identifying SNPs in putative promoter regions**. Cylinders represent databases. Rectangles are computational applications. (a) Putative promoter regions are identified in the human genome sequence. (b) Transcription binding sites are predicted in the putative promoter regions by using TransFac database. (c) SNPs are mapped. (d) Evolution conservation scores are calculated within transcription factor binding sites. (e) The disease association and functional annotation of target genes carried out by using an in-house functional annotation database.

We developed an integrated computational system for identifying SNPs in non-coding regulation regions (Fig 1). In this system, we: 1) predicted TF binding sites in putative promoter regions, 2) identified SNPs in the putative promoter regions and selected SNPs within predicted TF binding sites, 3) examined evolutionary conservation of predicted TF binding sites, and 4) integrated a variety of gene annotation information.

### Prediction of TF binding sites in putative promoter region

We identified TF binding sites in the putative promoter regions in the human genome. The promoter region is defined as the sequence of 5 kb upstream to 500 downstream bases of a transcription start site. The annotation information of genes, which is mapped to the genome, was obtained from the NCBI Gene database. To find TF binding sites in the putative promoter regions, we used the MATCH (Matrix Search For Transcription Factor Binding Site) program from the Transfac database (ver. 8.4) [18,19]. As a result, we predicted 1,497,317 TF binding sites from 28,644 human genes.

### Identification of SNPs on predicted TF binding sites

The SNP annotation information was derived from a public SNP database (dbSNP ver. 126). We identified SNPs in putative promoter regions and selected SNPs that are predicted to be within TF binding sites. As a result, we mapped 488,452 SNPs and filtered out 47,832 SNPs within the putative TF binding sites.

### Applying a conservation score

Using computational methods for predicting TFBS (TF binding sites) is not optimal due to a high false positive rate. However, recent algorithms have been improved in their reliability in TFBS prediction. Popular algorithms examine well-conserved regulatory sequences by comparing upstream sequences of orthologous genes across species [20-28]. Therefore, as an index of reliability for such an approach, we calculated an evolutionary conservation score for all the predicted TF binding sites. Users can see how reliable their predicted TF binding sites are. We used the phastcons16way file derived from UCSC human genome data. This file contains a conservation score from multiple genome alignment data calculated by the phastCons program [29].

### Integration with functional annotation

The SNP@Promoter database adopted various gene annotations including pathways (KEGG), gene ontology (GOA), and disease information such as GAD, HGMD, and OMIM. The raw data files were integrated into the SNP@Promoter database based on a gene synonym table from HGNC (HUGO). These annotations provide insight into the effects of SNPs within TF binding sites and help users to characterize target genes regulated by SNPs.

## User interface

**Figure 2:**
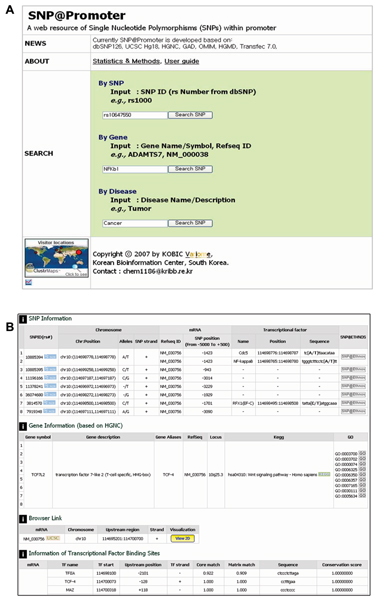
**SNP@Promoter user interface**. SNP@Promoter main page. (A) Users can search using three entries: 1) an SNP identifier (rs number from dbSNP), 2) a gene (Gene name, gene symbol, refSeq ID), or 3) a disease term. (B) SNP@Promoter gene retrieval page. The SNP Information table shows identified SNPs within putative promoter region and TF biding sites. The Gene Information table shows various gene annotations including pathways (KEGG), gene ontology (GOA). The Information of Transcription Factor Binding Sites table shows a variety off TF information such as TF start position, upstream position, TF strand, match score, TF binding sequences, conservations score.

**Figure 3:**
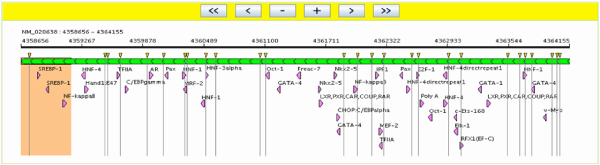
**A graphic viewer of transcription regulatory region**. The green bar represents a putative promoter region (5500 bp). The arrows in the green bar show a strand of transcription, orange box is transcription start region, yellow inverted triangles are SNP positions, and purple triangles are predicted transcription binding sites.

As shown in Fig. 2(A), a user can search the SNP@Promoter database using three kinds of entries: 1) an SNP identifier (rs number from dbSNP), 2) a gene (Gene name, gene symbol, refSeq ID), or (3) a disease term. When the user submits a gene or a disease term, SNP@Promoter returns a gene list related to queries. In the case of accessing details of the query gene, it shows SNP information, gene information, and transcription factor binding site information of target genes as shown Fig. 2(B). SNP@Promoter provides graphical views of the queried SNPs and genes. Fig. 3 shows a putative promoter region browser.

## Conclusion

SNP@Promoter is a database for functional SNPs within putative promoter regions and predicted TF binding sites. The database provides genetic information and graphical views of queried terms. SNP@Promoter will help researchers to identify functional SNPs in non-coding regions. Users can access the SNP@Promoter at  or directly at .

## Competing interests

The authors declare that they have no competing interests.

## Authors' contributions

BK constructed the database. WYK developed the website and assisted to construction of database. WH and KS helped to develop the website. BK initiated this project and wrote the manuscript. DP assisted the manuscript writing. JB directed the study and helped to draft the manuscript.
